# Endovascular stenting with a drug-eluting stent of transplanted renal artery stenosis in a dual kidney transplanted patient

**DOI:** 10.1590/1677-5449.210054

**Published:** 2021-11-29

**Authors:** Rajesh Vijayvergiya, Navjyot Kaur, Ganesh Kasinadhuni, Ashish Sharma, Anupam Lal, Ashwani Sood

**Affiliations:** 1 Post Graduate Institute of Medical Education and Research – PGIMER, Chandigarh, India.

**Keywords:** double kidney transplant, drug-eluting stent, end-stage renal disease, expanded criteria donor, percutaneous transluminal renal angioplasty, transplant renal artery stenosis, transplante renal duplo, stent farmacológico, doença renal em fase terminal, doador com critérios expandidos, angioplastia percutânea transluminal em artéria renal, estenose de artéria renal transplantada

## Abstract

Renal transplant remains the preferred therapy for end-stage renal disease (ESRD). Given the shortage of suitable donor kidneys, use of an expanded criteria donor (ECD) allows marginal kidneys to be transplanted; albeit at risk of increased graft failure due to lower nephron mass. To reduce the risk of graft failure, double kidney transplant (DKT) is advocated, with favorable outcomes. Transplant renal artery stenosis (TRAS) is one of the most common vascular complications following renal transplant. Unlike single kidney transplants, where TRAS usually presents with fluid overload, uncontrolled hypertension, and worsening kidney functions; it may be clinically silent in DKT patients since they have two functional transplanted kidneys. We hereby report a case of TRAS in a DKT patient who had 2 years of favorable clinical outcomes following successful endovascular stenting. He however recently died of COVID-19 associated pneumonitis.

## Resumo

O transplante renal continua sendo a terapia preferida para doenças renais em fase terminal. Dada a escassez de rins de doadores adequados, o doador com critérios expandidos permite que rins marginais sejam transplantados, embora haja um maior risco de falha do enxerto devido à diminuição da massa nefrótica. Para diminuir o risco de falha do enxerto, recomenda-se o transplante renal duplo (TRD), com resultados favoráveis. A estenose de artéria renal transplantada (EART) é uma das complicações vasculares mais comuns após o transplante renal. Ao contrário dos transplantes de rim simples, nos quais a EART geralmente se manifesta como sobrecarga de fluido, hipertensão descontrolada e piora das funções renais, ela pode ser clinicamente silenciosa em pacientes com TRD, pois eles têm dois rins funcionais transplantados. Relatamos aqui um caso de EART em um paciente com TRD que teve resultados clínicos favoráveis por dois anos após o sucesso do implante de stent endovascular. No entanto, ele morreu recentemente de pneumonite associada à covid-19.

## INTRODUCTION

With an ever-increasing number of patients with end-stage renal disease (ESRD), the demand-supply gap between those awaiting transplant and donor kidneys continues to widen. To overcome the shortage of donor kidneys, there has been a relaxation of donor criteria, known as expanded criteria donor (ECD), wherein less than the most suitable kidneys are accepted for transplant.^1^ To prevent graft failure due to reduced nephron mass of ECD kidneys, a double kidney transplant (DKT) has been advocated with favorable long-term graft function.^2^ DKT is the transplantation of 2 adult expanded criteria donor (ECD) kidneys into a single recipient to improve total nephron mass.^1^^,^ 2 We hereby report a case of transplant renal artery stenosis (TRAS) in a case of DKT, which was successfully managed by endovascular stenting.

## CASE REPORT

**Figure 1:**
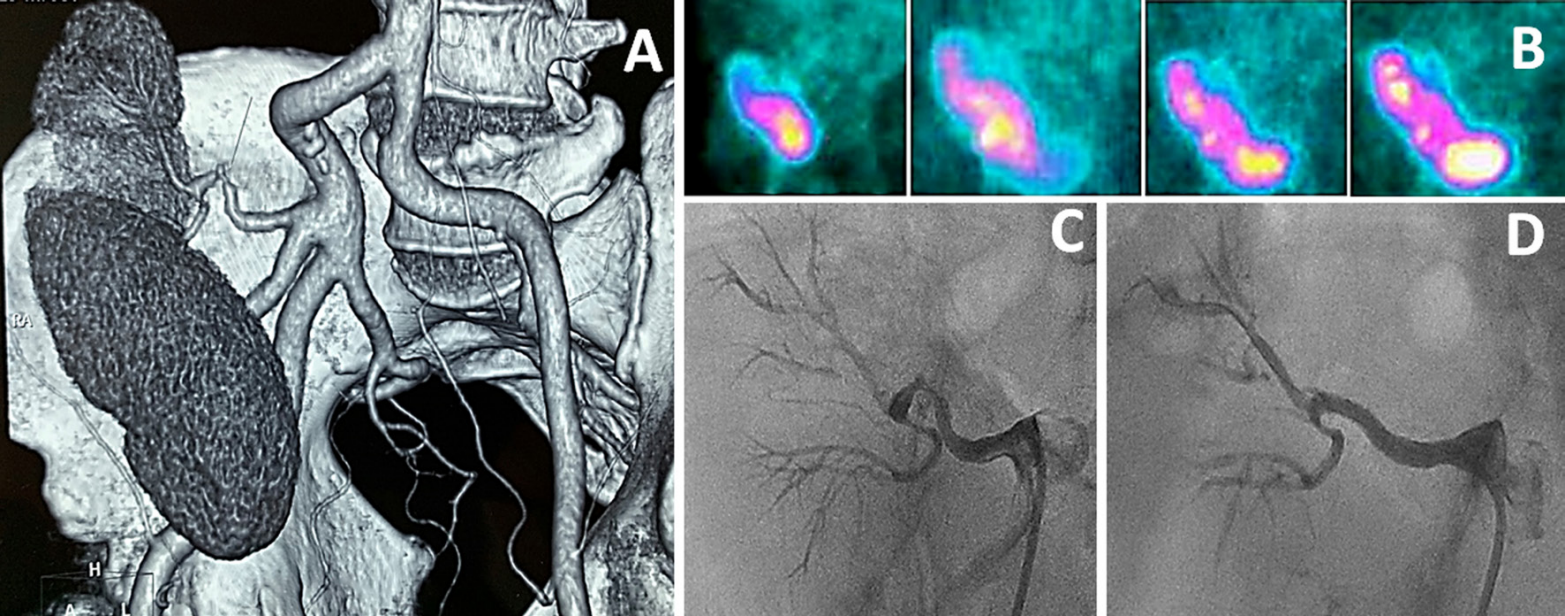
(A) Computed tomography (CT) image showed 80% stenosis of the transplanted renal artery (TRAS) of the posterosuperior kidney, while the renal artery of the anteroinferior kidney was normal; (B) A ^99m^Technitium diethylenetriamine-pentaacetate (DTPA) scan showed delayed radiotracer uptake by the cranially placed kidney, suggestive of TRAS; (C) A selective renal angiogram showed 80% stenosis of the transplanted main renal artery; (D) Following stenting, selective renal angiogram showed normal flow across the renal artery.

A 60-year-old male with ESRD underwent DKT from a 69-year-old ECD donor, after exhausting all options of getting a suitable criteria donor. The graft kidneys were harvested from the donor using standard organ procurement procedures and both the graft kidneys were transplanted into the right iliac fossa. The patient had an uneventful postoperative period and was discharged on immunosuppressive and antihypertensive therapy. His high blood pressure was controlled with Amlodipine 10 mg/day. He remained asymptomatic on follow-up with adequate blood pressure control and good urine output and had no fluid overload. His creatinine and estimated glomerular filtration rate were 0.80 mg/dL and 104.8 ml/minute/1.73m^2^ respectively. However, routine 3-month follow-up Doppler ultrasound revealed increased peak systolic velocity (PSV) and end-diastolic velocity (230 cm/sec and 165 cm/sec, respectively) across one of the transplanted renal arteries with renal aortic ratio (RAR) of 3.9, suggestive of TRAS. Computed tomography angiogram revealed 80% TRAS of the cranially placed transplanted kidney, while the renal artery of the caudally placed kidney was normal (Figure 1A). A ^99m^Technitium diethylenetriamine-pentaacetate (DTPA) scan showed delayed radiotracer uptake by the cranially placed kidney (Figure 1B). Both kidneys showed preserved perfusion and cortical tracer uptake with adequate clearance, suggestive of good functional status and viable affected kidney.

The patient was considered for endovascular stenting of TRAS. The affected renal artery was selectively cannulated with a 6-French (6F) Judkins Right-4 coronary guide catheter via right transfemoral access and an 80% stenosed lesion (Figure 1C) was crossed with a ChoicePT coronary guidewire (Boston Scientific, Natick, MA, USA). Following pre-dilatation with a 2.5 × 15 mm balloon, it was stented with a 3.5 × 23 mm everolimus drug-eluting coronary stent (Xience Prime stent, Abbott Vascular, Santa Clara, CA, USA). The proximal end of the stent was post-dilated with a 5 × 12 mm non-compliant balloon.

Good flow was achieved across the transplanted renal artery (Figure 1D). The patient was discharged on dual antiplatelet therapy along with immune-suppressive drugs. A repeat Doppler ultrasound showed normal flow across the intervened renal artery (PSV: 150cm/sec, end-diastolic velocity: 110 cm/sec, and RAR of 3.1), without any evidence of in-stent restenosis, at 1 year follow-up. His blood pressure remained under control with the same dose of amlodipine (i.e. 10 mg/day). He recently died due to COVID-19 associated pneumonitis after 2 years of follow-up. Informed written consent was taken for the percutaneous intervention of arterial disease. The case report was in accordance with the Helsinki Convention and approved by the institutional ethics committee for the retrospective evaluation.

## DISCUSSION

Kidney transplant remains the treatment of choice for patients with ESRD, considering the better quality of life and lower cardiovascular morbidity and mortality rates compared to other renal replacement therapies.^3^ With the increasing number of ESRD patients, the demand-supply gap between transplant recipients and donor kidneys has widened significantly.^4^ This has led to organs being taken from ECD patients, defined as donors over the age of 60 years without co-morbidities or donors between 50 to 59 years of age with any two co-morbidities such as hypertension, death from cerebrovascular accident, or terminal serum creatinine of >1.5 mg/dL.^1^ The advanced age and associated co-morbidities decrease the effective nephron mass and increase the risk of single kidney graft failure.^5^ To overcome this issue, transplantation of two kidneys from ECD to a single recipient (known as DKT) has been proposed and the results have been gratifying, especially for immediate graft function.^5^ Even at 5 years of follow-up, 50% of kidneys from ECD with DKT were functional as compared to 70% from suitable criteria donors.^2^

TRAS is a common vascular complication following renal transplant and usually occurs between 3 months to 2 years after transplant.^6^ Better immunosuppressive agents have significantly reduced the incidence of allograft rejection; making TRAS an important cause of graft loss.^7^ The site of TRAS may be the donor renal artery, at the suture site, or the recipient artery.^6^ Its etiology includes surgical suture technique, damage to vessel endothelium during graft harvesting or surgery, atheroma of the donor artery, external mechanical compression, and rarely due to immune-mediated injury.^8^^,^ 9 The exact cause of stenosis in the index case was not clear as the donor artery did not have any atheroma at the time of transplant and there were no apparent retrieval associated vascular injuries. The patient did not have any rejection to suggest immune-mediated injury. Unlike TRAS in a single kidney transplant, which presents with uncontrolled hypertension, fluid overload, and worsening renal parameters, our patient was well compensated with normal volume status and kidney functions and well-controlled blood pressure. A significant TRAS would increase renin levels and blood pressure, which would eventually compromise the function of both transplanted kidneys, the affected and the normal one, in the long term, as explained by Goldblatt’s “two-kidney, one-clip” model.^10^ Further, patients with transplanted kidneys usually develop iatrogenic hypertension following long-term use of immunosuppressive therapy, especially steroids and calcineurin inhibitors. DKT offers the advantage of extra nephrons to prevent late graft failure when ECD is used. Hence, any treatable cause compromising the function of either kidney should be intervened before irreversible damage, even if asymptomatic, which is contrary to the conventional strategy of only treating symptomatic TRAS in a single kidney transplant recipient.^11^^,^ 12

Timely detection of TRAS is important to preserve graft functions and we were able to detect it at 3 months of follow-up. We routinely perform screening ultrasound in all post-transplant patients at 3 months of follow-up to detect TRAS. Doppler ultrasound is the initial diagnostic and screening modality for diagnosing TRAS.^12^ Increased PSV (> 180 cm/sec), end-diastolic velocity (> 150 cm/sec), and RAR > 3.5 are suggestive of RAS; in addition, pulsus parvus et tardus, slow systolic upstroke, and an increase in acceleration time are useful indicators of significant stenosis of the renal artery.^13^^-^ 15 Percutaneous renal angioplasty with stenting remains the treatment of choice for TRAS.^16^^-^ 18 A common complication of renal artery stenting is the in-stent restenosis of bare-metal stents, especially if the target vessel diameter is less than 5 mm.^19^ Since our case had a smaller target artery, we used a 3.5 mm drug-eluting stent, which has shown a lower in-stent restenosis rate compared to bare metal stents.^20^ To the best of our knowledge, we could not find any published cases in the English literature of TRAS in a DKT patient successfully treated with endovascular stenting.

In conclusion, we hereby describe a case of DKT in a patient who had TRAS of one of the transplanted kidneys. He had 2 years of favorable clinical outcomes following successful endovascular stenting of TRAS.
